# An Unusual Case of Syphilis With Pulmonary Involvement

**DOI:** 10.7759/cureus.63758

**Published:** 2024-07-03

**Authors:** Hadia Arzoun, Fawaz Mohammed, Subha Saeed, Tahir Muhammad Abdullah Khan, Karan Singh

**Affiliations:** 1 Pulmonary and Critical Care Medicine, University of Kentucky College of Medicine, Bowling Green, USA; 2 Internal Medicine, St. Bernard's Medical Center, Jonesboro, USA; 3 Internal Medicine, California Institute of Behavioral Neurosciences and Psychology, Fairfield, USA; 4 Internal Medicine, University of Kentucky College of Medicine, Bowling Green, USA; 5 Internal Medicine, Marshfield Medical Center, Marshfield, USA

**Keywords:** treponema pallidum, penicillin g, right-sided pleural effusion, human immuno deficiency virus, atypical syphilis

## Abstract

Syphilis can affect multiple organs in the secondary or tertiary stages of the disease. Recent reports have suggested an increase in the incidence of the disease. Involvement of the lung has been rarely described in syphilis. In this report, we discuss the case of a 26-year-old female with past medical history significant for HIV who presented to the hospital with complaints of shortness of breath and underwent thoracentesis; she was found to have syphilis with pulmonary involvement.

## Introduction

Syphilis is a sexually transmitted disease, and *Treponema pallidum* is the organism responsible for it. It is transmitted through sexual contact and manifests with an array of signs and symptoms depending on the stage of the disease. Other routes of infections are infection in utero, blood transfusions, or organ transplantation [1]. The number of infections has dropped drastically since the 1940s thanks to the use of penicillin [1]. The disease course commences with an incubation period ranging from three to six weeks. The disease itself has four stages (primary, secondary, tertiary, latent), with each stage having its distinctive characteristics. Only a handful of cases with pulmonary syphilis have been described in the literature, with the majority of cases reported in the preantibiotic era [2]. Pulmonary involvement was more commonly seen in tertiary and congenital syphilis, although it is more often seen in secondary syphilis after the discovery of antibiotics.

We present a case of a 26-year-old female with past medical history significant for HIV on treatment with bictegravir/emtricitabine/tenofovir alafenamide. She presented to the hospital on account of worsening shortness of breath. Chest imaging showed evidence of a pleural effusion, with pleural fluid cultures subsequently yielding *Treponema pallidum *species.

## Case presentation

A 26-year-old female with a recent diagnosis of syphilis who was to commence treatment presented to the hospital with complaints of worsening shortness of breath, fevers, and diarrhea. She denied chest pain, palpitations, nausea, vomiting, or abdominal pain. She also complained of a cough that was non-productive in nature. Her past medical history was significant for HIV and she was on bictegravir/emtricitabine/tenofovir alafenamide. Her recent laboratory values from the outpatient setting showed undetectable HIV RNA with a CD4 count of over 700 (500 to 1500 cells/mm^3^). Blood pressure on presentation was 151/94 mmHg, and she had a heart rate of 109/min. She was found to be hypoxic, requiring supplemental oxygen. The complete blood count at the time of presentation is presented in Table 1.

**Table 1:** Complete blood cell count on presentation

Variables	Patient values	Reference values
White blood cell count (k/ul)	16.7	4.8-10.8
Hemoglobin (g/dl)	9.4	12-16
Hematocrit (%)	31.9	34-47
Platelet levels (k/ul)	556	140-440

**Figure 1:**
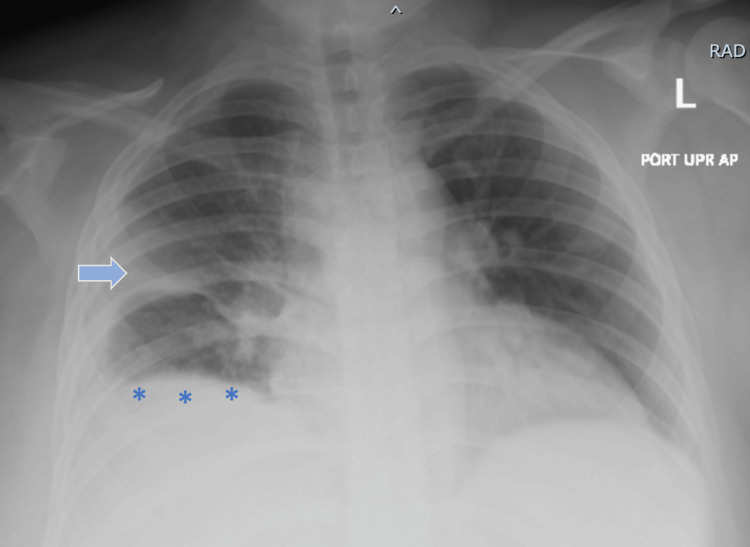
Chest X-ray findings Chest X-ray demonstrating right middle lobe infiltrate (blue arrow) and right-sided pleural effusion (asterisks)

Chest X-ray showed evidence of a right middle lobe infiltrate and pleural effusion (Figure 1).

**Figure 2:**
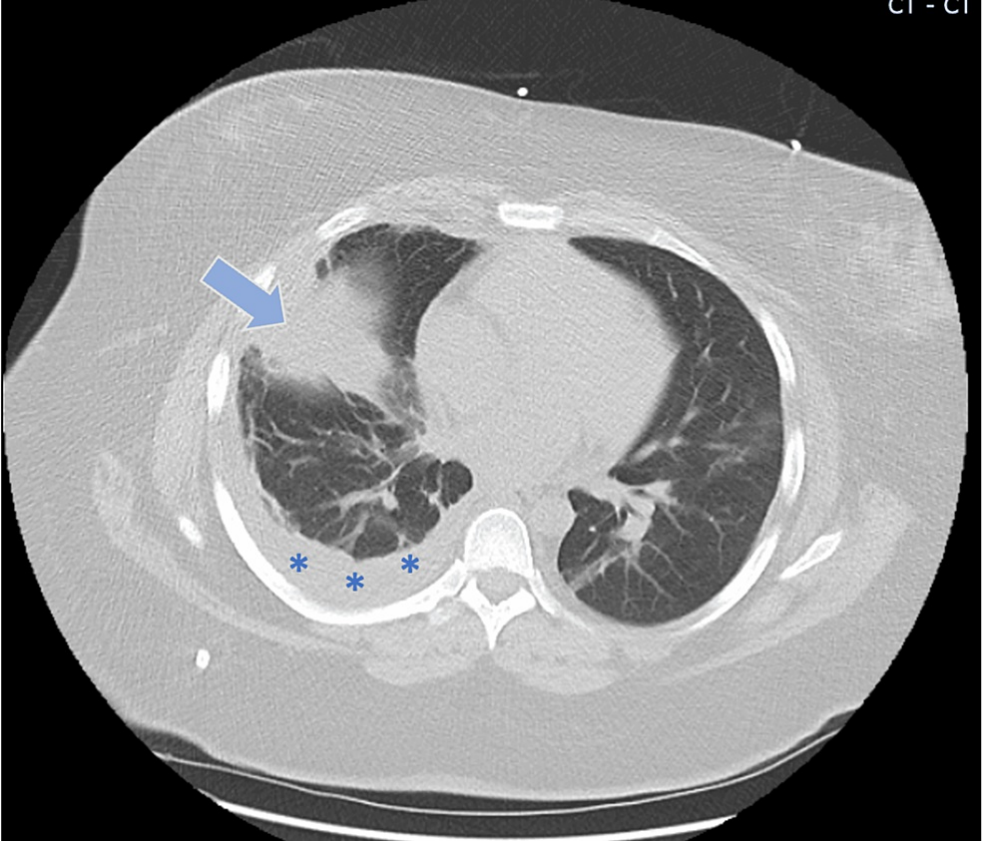
Chest CT findings Chest CT with right middle lobe infiltrate (blue arrow) redemonstrated, also confirming the presence of pleural effusion on the right side (asterisks) CT: computed tomography

For further evaluation, a chest CT was performed, which confirmed the pleural effusion and infiltrate in the right middle lobe (Figure 2). Pulmonology was consulted and a thoracentesis was then performed. Results from pleural fluid studies are presented in Table 2. 

**Table 2:** Results of pleural fluid analysis

Variables	Pleural fluid parameters	Reference values
Appearance	Dark-amber colored	-
Total protein (g/dl)	4.9	1-2
Glucose (mg/dl)	110	90-120
Lactate dehydrogenase (U/L)	1600	40-280
White blood cell count (u/l)	49	0-5
Neutrophils (%)	1	0-100
Lymphocytes (%)	17	0-100

**Figure 3:**
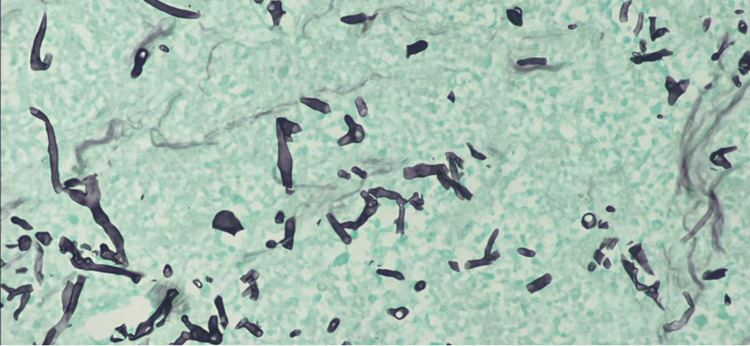
Immunohistochemical stain of pleural fluid Immunohistochemical stain for *Treponema pallidum* showing multiple spirochetes

The pathology report from pleural fluid was positive for *Treponema pallidum* species on immunohistochemical stain (Figure 3). Infectious disease was consulted and the patient was discharged in stable condition with a planned treatment course of three weeks with intravenous penicillin for syphilis complicated by empyema.

## Discussion

Our review of the literature has indicated that lung involvement with syphilis is rather rare [1-3]. Other sites less frequently involved include the brain, eye, ear, and gastrointestinal organs [4]. On chest imaging, pulmonary syphilis can present as bilateral infiltrates, single or multiple nodules, or, in rare cases, pleural effusion as seen in our case [5]. A review by Youssef et al. reported co-infection with HIV is seen in about 18% of the patients [6]. These findings coincide with different disease processes often delaying the diagnosis. Therefore, the following criteria proposed by Coleman et al. can be utilized to aid diagnosis: (i) historical and physical findings typical of secondary syphilis; (ii) serologic test results positive for syphilis; (iii) pulmonary abnormalities seen radiographically with or without associated symptoms or signs; (iv) exclusion of other forms of pulmonary disease, when possible, according to findings of serological tests, sputum smears and cultures, and cytological examination of sputum; and (v) response to anti-syphilis therapy [7].

Given that *Treponema pallidum *is difficult to culture, it may be diagnosed with serology [8]. Standard serological testing consists of a non-treponemal test, which is then confirmed with fluorescent treponemal antibody absorptiometry [9]. Clinicians need to be mindful that false-positive treponemal tests are a possibility; these are seen in infections caused by other spirochetes or even malaria [10]. The discrepancy between the nontreponemal and treponemal test may also be observed in patients previously treated for syphilis, in very early or late stages of the disease when the non-treponemal test is essentially non-reactive or in individuals with immunocompromised states who cannot mount an appropriate B-cell response [11-13]. Polymerase chain reaction (PCR) on bronchoalveolar lavage (BAL), pleural fluid, lung biopsy, and immunohistochemistry may also be of benefit to identify the indicated spirochete [6].

Penicillin is the antibiotic of choice for all stages of the disease [14]. One dose of intramuscular benzathine penicillin G 2.4 million units suffices for primary, secondary, and early latent stages of the disease. Three doses of the same are required at weekly intervals in late latent and tertiary syphilis. Doxycycline, a third-generation cephalosporin like ceftriaxone, and ampicillin in combination with probenecid may be used in patients unable to tolerate penicillin [15].

## Conclusions

We presented a rare case of syphilis with pulmonary involvement. Clinicians need to be aware of this type of atypical involvement in syphilis. In individuals presenting with airspace disease in the lungs along with pleural involvement, the differential diagnosis is often wide. With epidemiological data suggesting that syphilitic cases are on the rise, a high index of suspicion for pulmonary syphilis should be maintained when investigating the more common causes of such clinical presentations.
